# Items of General Interest

**Published:** 1915-11

**Authors:** 


### Items of General Interest.

An interesting program was rendered by the Hunt County Medical Society at its meeting the latter part of September.

The regular semi-annual meeting of the Eleventh District Medical Society was held in Palestine the latter part of September, the program consuming all of one day.

The Milam County Medical Society met in regular quarterly session in Rockdale recently. The program was cut short on account of the absence of several members who were to have read papers.

The annual meeting of the members of the West Texas Medical Society was held November 2 and 3. On the evening of November 3 a meeting was held at the Texas Tuberculosis Sanitarium at Carlsbad, fifteen miles from San Antonio.

The physicians of Cleburne have organized the Doctors' Business League, with Dr. J. D. Osborn president and Dr. W. R. Washburn secretary. The purpose of the organization is the mutual protection of the members and aiding in the elimination of bad debts.

According to a statement by the London School of Medicine for Women, the demand for women doctors exceeds the supply. A position in the Whitechapel district, held before the war by a man at a salary of $500 a year and emoluments, is now advertised for a woman at $1000 and emoluments.

John Brennan, the managing editor of *Physical Culture*, announces a series of articles on birth control. These articles have been prepared by one of the world's famous physicians, Dr. Havelock Ellis of London. These articles will appear in September, October, and November issues of *Physical Culture*. We commend them to anyone desiring information on birth control.

A well attended and interesting meeting of the Victoria-Calhoun County Medical Society was held at Victoria during the latter part of September. Dr. J. L. Smith presented a clinical case of cancer, the history and treatment of which was of much interest to those present.

Dr. E. A. Malsch presented a clinical case of femoral aneurism, now cured. Both of these cases were most unusual, and excited much interest and discussion.

---

The following program was rendered at a meeting of the McLennan County Medical Society at Waco on October 3:

"Skin Cancer," Dr. W. L. Crosthwait, Waco.

"Neurotic Dermatosis," Dr. Guy F. Witt, Waco.

"Tubercular Cutis," Dr. R. H. Dudgeon, Waco.

"Eruptive Fever, Dr. J. G. Wigham, Moody.

"Skin Lesions in Syphilis," Dr. I. L. McGlasson, Waco.

Report of clinical cases.

---

The Medical branch of the University of Texas opened for registration of students in September with a splendid number in attendance, many new students having registered, as well as those who have come to continue their work from last year. An interesting feature of the opening exercises was an address by Dr. Allen G. Heard, adjunct professor of medicine, on "Diseases of Children." Following Dr. Heard's address, Dr. W. J. Battle, acting president of the University of Texas, was introduced by Dr. W. S. Carter, dean of the medical college faculty, and addressed the college.

---

*The Journal of Laboratory and Clinical Medicine* has made its first bow to the medical profession in the October issue. Dr. Victor C. Vaughn is the editor in chief. This alone should accord it a warm welcome. It is intended that this journal shall represent the best medical thought in America, and the editorial staff justifies the belief that it will. The journal is published by the C. V. Mosby Co. of St. Louis, and anyone who has seen the *Journal of Orthodontia,* or read books published by this firm, knows they spare no pains or expense to make their publications attractive, interesting, and helpful.—Ed.

---

The Texas Surgical Society met in San Antonio October 18th and 19th. The attendance was splendid, eighteen out of the twenty members being present. The papers were very excellent and the discussions proved very helpful. Many of the San Antonio physicians took advantage of the meeting.

On the evening of the 18th Dr. and Mrs. Venables honored the

Society with a reception in their beautiful home. It was a charming affair, and the doctor and his beautiful wife proved themselves most gracious hosts. Everybody had a good time, and the visiting physicians expressed themselves as delighted to have an opportunity to greet old friends and make new acquaintances.

---

At the last meeting of the Southwestern Medical Association, which was held in Oklahoma City October 12th and 13th, Dr. Joe Becton, of Greenville, Texas, was elected President. The Association is composed of men from Texas, Oklahoma, Arkansas, Kansas and Louisiana. It is a very distinguished honor to be selected President of such an Association, and Dr. Becton has won another laurel for Texas. The other officers selected are as follows: Walter S. Sutton, Kansas; J. H. Riddel, Arkansas; E. H. Martin and St. Cloud Cooper, Oklahoma; J. A. Walker and W. A. Ball, Missouri, and S. C. James and E. H. Skinner of Texas, Vice-Presidents.

---

Baylor University Medical College has opened its classes for the sixteenth session. Addresses by leading church and medical men preceded the routine of work. The opening exercises included addresses by Dr. George W. Truett, Dr. S. P. Brooks, president of Baylor University at Waco; Dr. J. H. Gambrell, Dr. E. H. Cary, dean of the college; Prof. E. G. Eberle, dean of the pharmacy department; Dr. S. E. Milliken of the Board of Health of Dallas, and Dr. T. G. Crowe, president of the State Board of Medical Examiners.

Dr. Milliken stated that Dallas has admirable facilities for a medical school, and congratulated the institution on its opportunities from this standpoint.

Dr. Cary referred to Dr. Milliken's talk, and said Dallas had more hospital-trained men in its medical fraternity than any city of its size anywhere. The existence of only one medical school in Dallas brought congratulations from Dr. Crowe, who said it is the confirmation of an idea he has had in mind for many years.

---

### School Exhibit on Prevention of Disease.

A circulating exhibit on Prevention of Disease for the Public Schools has been prepared by Dr. W. A. Davis, secretary of the State Board of Health. The exhibit is enclosed in a case and contains ten charts, 22x28 inches, with a booklet of ten lectures on the subjects pictured on the charts. It is intended that the exhibit be sent to the school, and the teacher will carry the lecture back to her room, read the lecture, and with her fund of general information teach the pupil the lesson, using the chart for the lecture as a text. The exhibit is then closed until the next day, and the second day's subject is handled in the same way, and so on for ten days or two weeks. The exhibit will then be sent to the next school. At the beginning of each course a prize could be offered for the best essay written on any subject treated and the work made to fit in with the regular school work. The local health officer or physician may be induced to lecture to the pupils, and in this way increase the interest.

The following subjects are treated in the charts:

(1) Home Sanitation—The Model Home. The House as it is.

(2) Stimulants—Tea, coffee, tobacco, alcohol.

(3) Personal Hygiene—Morning bath, the shoe, the clothing, the eye, the teeth, the hand, the diet, the sleeping room.

(4) Accidents—Burns, nail punctures, bites and stings, hydrophobia, drowning and artificial respiration.

(5) Our Uninvited Guests—Flies, fleas, bedbugs, mosquitoes, lice.

(6) Preventable Diseases—Diphtheria, meningitis, lockjaw, typhoid fever, tuberculosis, the sanitary and unsanitary sick room.

(7) Preventable Diseases—Adenoids, protection of the neighborhood, protection of the home.

(8) Vital Statistics—Birth registration, notifiable diseases, death records, smallpox.

(9) School Sanitation—The school grounds, the desk, light, heat and ventilation, water supply, dry sweeping, the well.

(10) Social Hygiene—The star of honor, the single standard.

Short, concise, pointed expressions are placed on the charts. The language used is common everyday English. No technical or scientific terms are found in the lectures. The pictures on the charts are placed in opposition as far as possible so as to call attention to the difference of the right and wrong involved in the subject. They are so plain that the pupil in the primary grades may understand the lesson to be taught. All statistics and data are given in the lecture so that the teacher may fit the lesson to the grades she is teaching, and the exhibit may be used in all grades from the primary to the fourth year of high school, and even by speakers in popular lectures on preventive medicine.

This is a splendid idea and Dr. Davis deserves much credit for having made it so helpful and so simple that the smallest child may understand.—Ed.

The costumer came forward to attend to the nervous old beau who was mopping his bald and shining poll with a big silk handkerchief.

"And what can I do for you?" he asked.

"I want a little help in the way of a suggestion," said the old fellow. "I intend going to the French students' masquerade ball tonight, and I wanted a distinctly original costume, something I may be sure no one else will wear. What would you suggest?"

The costumer looked him over attentively, bestowing special notice on the gleaming nob.

"Well, I'll tell you," he said, then thoughtfully, "why don't you sugar your head and go as a pill?"

---

"Some men have no hearts," said the tramp. "I've been a-tellin' that feller I am dead broke, that I have to sleep outdoors."

"Didn't that fetch him?" asked the other.

"Naw. He told me he was a-doin' the same thing, and had to pay the doctor for tellin' him to do it."—*Christian Register.*

---

FOLLOWED INSTRUCTIONS.

"Gracious, man!" exclaimed the doctor, when Mr. Glubbins calls him in a hurry, "your temperature is rioting along near the danger point, and you—"

"And I'm worse off than I ever was before, all through the diet you prescribed."

"Impossible, Mr. Glubbins. I told you distinctly to confine yourself to such food as would be taken by a three-year-old child."

"And didn't I obey orders? I ate apple cores, dog biscuits and ends of burned matches and scraps of peelings and everything else I could pick up while no one was looking, and here I am pretty nearly dead."

Hastily reflecting upon the gastronomical tendencies of the average three-year-old child, the doctor tells Mr. Glubbins that he has been overdoing the diet and will have to subsist on soft toast and hot water for a week.—*Philadelphia Public Ledger.*

---

The first annual meeting of the National Committee for the Prevention of Blindness will be held in the assembly room of the Russell Sage Foundation Building, corner of East Twenty-second Street and Lexington Avenue, New York, at 4:30 p. m., November 4th. Ex-President William H. Taft will be present and will speak, and Dr. G. E. de Schweinitz, of Philadelphia, will make an address.

Persons wishing to avail themselves of the exhibits should take pains to make early request so that if there are not enough exhibits to meet requirements new ones can be prepared. Send us the name of the chairman of the health committee of your State or county fair association and we will be glad to tell him how he can secure exhibits on the prevention of blindness.

In the vacation months, a large number of the committee's publications were distributed during the meetings of various societies and organizations in San Francisco, California; Newport, Rhode Island; Grand Rapids, Michigan; Chicago, Illinois; and Milwaukee, Wisconsin.

While ophthalmia neonatorum (babies' sore eyes) continues to send to the schools for the blind a large percentage of unfortunate children (nearly 100 in the school year 1914-15), a gratifying reduction is shown by comparing the reported percentages for the five years since 1910. Of the new pupils entering in each of the following years the per cent blind from babies' sore eyes is:

| | |
|---|---|
| 1910-11 | 23.9 per cent. |
| 1911-12 | 21.2 per cent. |
| 1912-13 | 22.7 per cent. |
| 1913-14 | 19.6 per cent. |
| 1914-15 | 15.1 per cent. |

More care in making reports and greater accuracy in diagnosis are probably responsible for some of the reduction indicated, but it is believed that the agitation which has been conducted by the various committees, commissions and societies for prevention of blindness is beginning to bear fruit.

The *Journal of the American Medical Association* has opened a campaign to secure the prohibition of the manufacture of methyl alcohol. commonly known as "wood alcohol." The extreme danger attending the use of methyl alcohol, even when confined to external application, and particularly the danger of confusing the poisonous wood alcohol with grain alcohol, is the animus inspiring the *Journal's* editorial campaign, in which, as the mouthpiece of the American Medical Association, it must have the backing and co-operation of the members of the association. Recently three persons died and two others were made completely blind from drinking a cordial made partly of wood alcohol, says the *Journal of the American Medical Association.*

